# Differential effect of parity on rat mammary carcinogenesis after pre- or post-pubertal exposure to radiation

**DOI:** 10.1038/s41598-018-32406-1

**Published:** 2018-09-25

**Authors:** Masaru Takabatake, Kazuhiro Daino, Tatsuhiko Imaoka, Benjamin J. Blyth, Toshiaki Kokubo, Yukiko Nishimura, Kaye Showler, Ayaka Hosoki, Hitomi Moriyama, Mayumi Nishimura, Shizuko Kakinuma, Masahiro Fukushi, Yoshiya Shimada

**Affiliations:** 1 Department of Radiation Effects Research, National Institute of Radiological Sciences (NIRS), National Institutes for Quantum and Radiological Science and Technology (QST), Chiba, 263-8555 Japan; 2 Department of Radiological Sciences, Graduate School of Human Health Sciences, Tokyo Metropolitan University, Tokyo, 116-8551 Japan; 3 Present Address: Division of Radiation Oncology and Cancer Imaging, Peter MacCallum Cancer Centre, Melbourne, Victoria 3000 Australia; 4 Department of Engineering and Safety, NIRS, QST, Chiba, 263-8555 Japan; 5 Present Address: Department of Radiology, The Jikei University Hospital, Tokyo, 105-8471 Japan; 6 Present Address: Healios K.K. Kobe Research Institute, Kobe, 650-0047 Japan; 7 Executive Director, QST, Chiba, 263-8555 Japan

## Abstract

Radiation exposure during the peri-pubertal period is a proven risk factor for breast cancer, whereas parity is an established protective factor. The present study investigated whether parity imposes differential protective effects against radiation-induced rat mammary carcinoma depending on the age at exposure. Pre- and post-pubertal female rats, irradiated or left unirradiated, were mated and allowed to nurse until weaning or left unmated. Appearance of mammary tumors was monitored, and serum concentrations of estradiol and progesterone were measured following weaning. Carcinomas were evaluated by immunohistochemistry for estrogen receptor, progesterone receptor, and the cell proliferation marker Ki-67. Parity reduced the risk of carcinoma in unirradiated and pre-pubertally irradiated rats but not post-pubertally irradiated rats. Although radiation exposure increased serum progesterone level, parity after pre-pubertal exposure significantly decreased the elevated progesterone to a normal level, reflecting a protective effect. Moreover, parity significantly decreased the proportion of hormone receptor–positive carcinomas after pre-pubertal exposure. Parity was also related to the observed positive association between progesterone receptor and Ki-67 indices in cancer tissue, implying progesterone receptor–dependent cell proliferation. Thus, parity protects against radiation-induced rat mammary carcinogenesis depending on the age at exposure; the mechanisms may involve changes in hormone levels and cancer tissue.

## Introduction

Breast cancer is the most common cancer for women in both developed and developing countries^1^. Epidemiologists have identified several risk factors for breast cancer such as diet and reproductive history^2,3^, with exposure to ionizing radiation from both medical and accidental exposures a proven risk factor^4,5^. The occurrence of breast cancer as a second cancer is of great concern for women who received radiation therapy in childhood, with a longer timeframe in which to manifest a radiation-induced cancer than for those treated as adults^6^. Mammary glands are thought to be highly susceptible to radiation-induced cancer around the time of puberty because of the onset of rapid growth^7^. In addition to this age factor, population data suggest that lifestyle, diet, and reproductive history act as risk modifiers for both spontaneous and radiation-induced breast cancer^4^. Epidemiological studies have documented that pregnancy and lactation at an early age reduces a woman’s lifetime risk of breast cancer^8^, and studies of Japanese atomic-bomb survivors have also shown that pregnancy decreases the radiation-related risk of breast cancer^9,10^. However, the risk of Hodgkin’s lymphoma following radiotherapy is not reduced by parity^11^, suggesting that the effects of parity on radiation-induced cancer are complex and potentially site-specific. This complexity is further underscored by a study of childhood cancer survivors, which revealed that breast cancer risk was sharply reduced among women who received radiation therapy as a child with a high dose of exposure to the ovaries^5^.

Rodent models have been widely used to study the protective effects of parity with respect to mammary tumorigenesis^12^, including thorough investigations of parity and chemically induced mammary carcinoma^13,14^. Rat models have been used to study the risk and underlying mechanisms of both chemically and radiation-induced mammary cancer because these models mimic both the pathogenesis of human breast cancer and the expression of hormone receptors (HRs), such as the estrogen receptor (ER) and progesterone receptor (PR), in diseased tissue^15^. To our knowledge, however, only one study has assessed the effects of radiation in female rats prior to pregnancy^16^. Therefore, the combined effects of parity and radiation on the risk of mammary cancer remain unknown.

Several mechanisms underlying the protective effect of parity against breast cancer have been proposed^8,17^. Some epidemiological studies have reported that parity reduces the risk of HR-positive, but not HR-negative, breast cancer^18–20^. Several lines of evidence have also shown that both parity and exposure to radiation alter the levels of certain circulating hormones such as estradiol and growth hormone^21–24^. However, the observed changes in hormonal status have not been consistently reproduced in experimental settings, and thus it remains unclear how radiation exposure affects hormonal status during tumorigenesis.

We previously reported that pre-pubertal, but not post-pubertal, radiation exposure induces premature cessation of the regular estrous cycle^25–27^, consistent with the documented effects of radiation on ovarian function^5^ and the fact that tumor subtypes differ between mammary carcinomas induced by pre- or post-pubertal radiation exposure^27^. Also, certain experimental and computational studies have addressed the mechanisms underlying the difference between the effects of radiation exposure during pre- and post-pubertal stages^28,29^. However, it is unknown whether there is an interaction between the age at radiation exposure and parity with respect to the risk of mammary cancer. Understanding any combined effects of age at radiation exposure and parity may improve the accuracy of predicting and controlling the risk of second breast cancer after radiation therapy in childhood. Moreover, such an understanding would provide a firmer biological basis on which to model radiation-induced cancer risk in future epidemiology analyses.

Given the aforementioned lines of evidence, we hypothesized that the effects of parity on radiation-induced mammary cancer would differ with respect to pre- or post-pubertal irradiation because age at exposure (pre- vs. post-pubertal) has been shown to affect the HR status of radiation-induced tumors. We therefore examined whether the effect of parity on the risk of mammary cancer depends on the age at which rats were exposed to radiation. Our findings indicate that parity indeed has differential effects on mammary cancer risk posed by pre- or post-pubertal radiation exposure; parity reduced the development of HR-positive tumors in rats irradiated before puberty, but not after. Postulated mechanisms underlying the age effect include changes in hormonal and tumor status, involving progesterone level and consequent proliferation of cancer cells, in parous rats.

## Results

### Differential effect of parity on rat mammary carcinogenesis induced by pre- or post-pubertal radiation exposure

**Figure 1:**
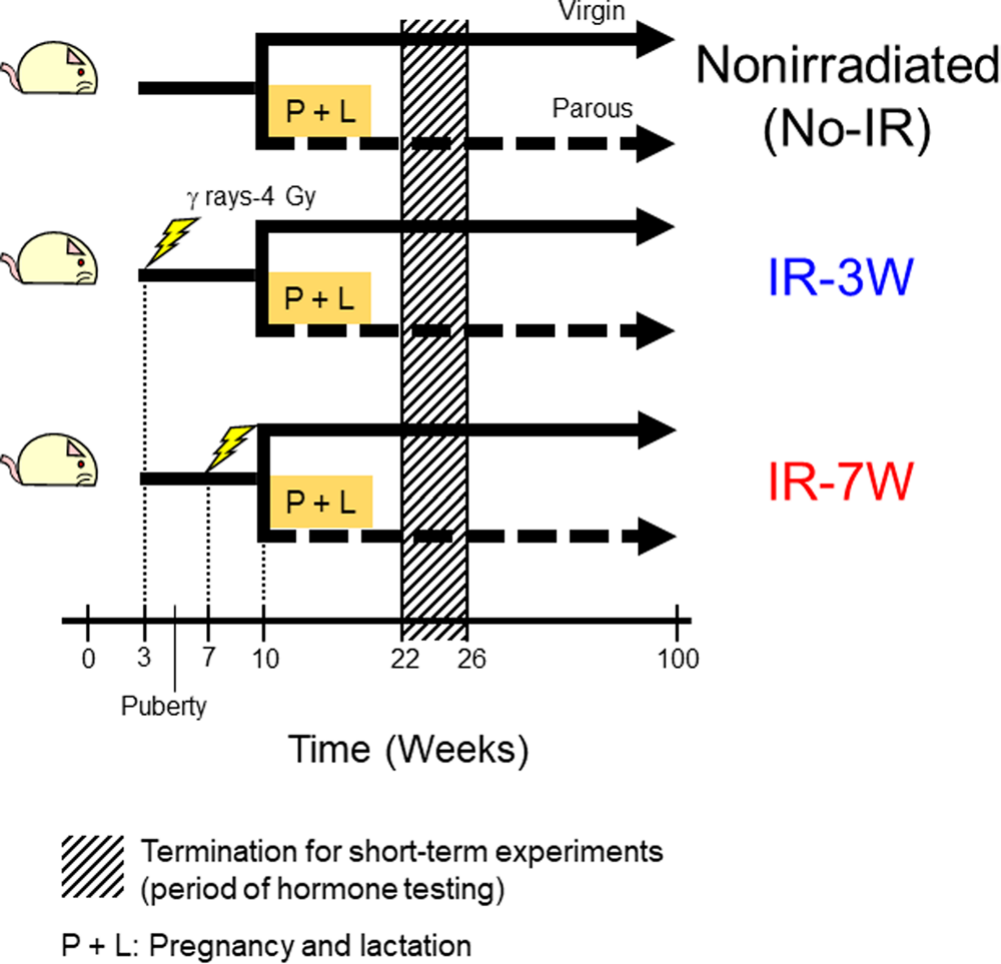
Schematic overview of the animal experiment. Pre- or post-pubertal female rats (3 or 7 weeks of age, respectively) were irradiated with 4 Gy of γ rays from ^137^Cs or left untreated. Half of the rats in each group were mated at 10 weeks of age. Mated rats that completed delivery, lactation (for 3 weeks), and weaning by the time they were 18 weeks old were used as parous rats. The experiment for evaluating mammary tumor risk was terminated at age 100 weeks. To assess hormonal status during the period of tumorigenesis separate experimental groups were established in the same manner. Parous rats in proestrus were autopsied at 5 weeks after weaning (age 22–26 weeks), and likewise for age-matched virgin rats. In the figure, the period denoted by hatched shading indicates the termination of the short-term experiment for the hormone tests. P + L, pregnancy and lactation; IR-3W, irradiation at age 3 weeks (pre-puberty); IR-7W, irradiation at age 7 weeks (post-puberty); No-IR, nonirradiated control; each group comprised virgin and parous subgroups.

To investigate possible interactions between the age at ionizing radiation (IR) exposure and parity with respect to the development of rat mammary tumors, we established six experimental groups: pre-pubertal irradiation (IR-3W), post-pubertal irradiation (IR-7W), and nonirradiated (No-IR), with virgin and parous groups for each. The experimental procedure is schematically shown in Fig. 1. The percentage of rats that ultimately developed mammary carcinoma by age 100 weeks was not significantly different between the parous rats and the exposure-matched virgin groups (Table 1). However, the rate of carcinoma manifestation (the mean number of postmortem-confirmed carcinomas detected per week) was significantly decreased by parity in the IR-3W group. In addition, the mean time to first palpation of mammary carcinoma was significantly delayed by parity in both the IR-3W and No-IR groups. The incidence, rate, and latency of mammary carcinomas were unaffected by parity in the IR-7W group.

**Table 1:** Summary of mammary tumorigenesis in virgin and parous rats.

Age at exposure (week)	Parity	Rats with tumor (%)	Tumors observed per week (10^−2^)	Age at first palpation (week)
Carcinoma				
—^a^	Virgin	11/26 (42 ± 10)	2.1 ± 0.5	60 ± 15
	Parous	7/28 (25 ± 8)	1.5 ± 0.2	90 ± 6^**^
3	Virgin	15/26 (58 ± 10)	4.5 ± 0.9	49 ± 13
	Parous	17/31 (55 ± 9)	1.9 ± 0.3^*^	66 ± 13^***^
7	Virgin	21/26 (81 ± 8)	3.5 ± 0.6	45 ± 19
	Parous	21/28 (75 ± 8)	3.2 ± 0.5	43 ± 22
Benign tumor (fibroadenoma/adenoma)				
—	Virgin	16/26 (62 ± 10)	2.9 ± 0.4	69 ± 18
	Parous	20/28 (71 ± 9)	3.0 ± 0.4	76 ± 17
3	Virgin	22/26 (85 ± 7)	5.2 ± 0.6	49 ± 14
	Parous	29/31 (94 ± 4)	5.4 ± 0.6	59 ± 17^***^
7	Virgin	21/26 (81 ± 8)	5.2 ± 0.5	53 ± 15
	Parous	25/28 (89 ± 6)	5.3 ± 0.6	58 ± 17

^a^Nonirradiated. *p < 0.05; **p < 0.005; ***p < 0.001 vs. matched virgin (i.e. same exposure treatment) by Mann-Whitney’s *U* test. Percentage and tumor number are mean ± SE; age at palpation is mean ± SD.

**Figure 2:**
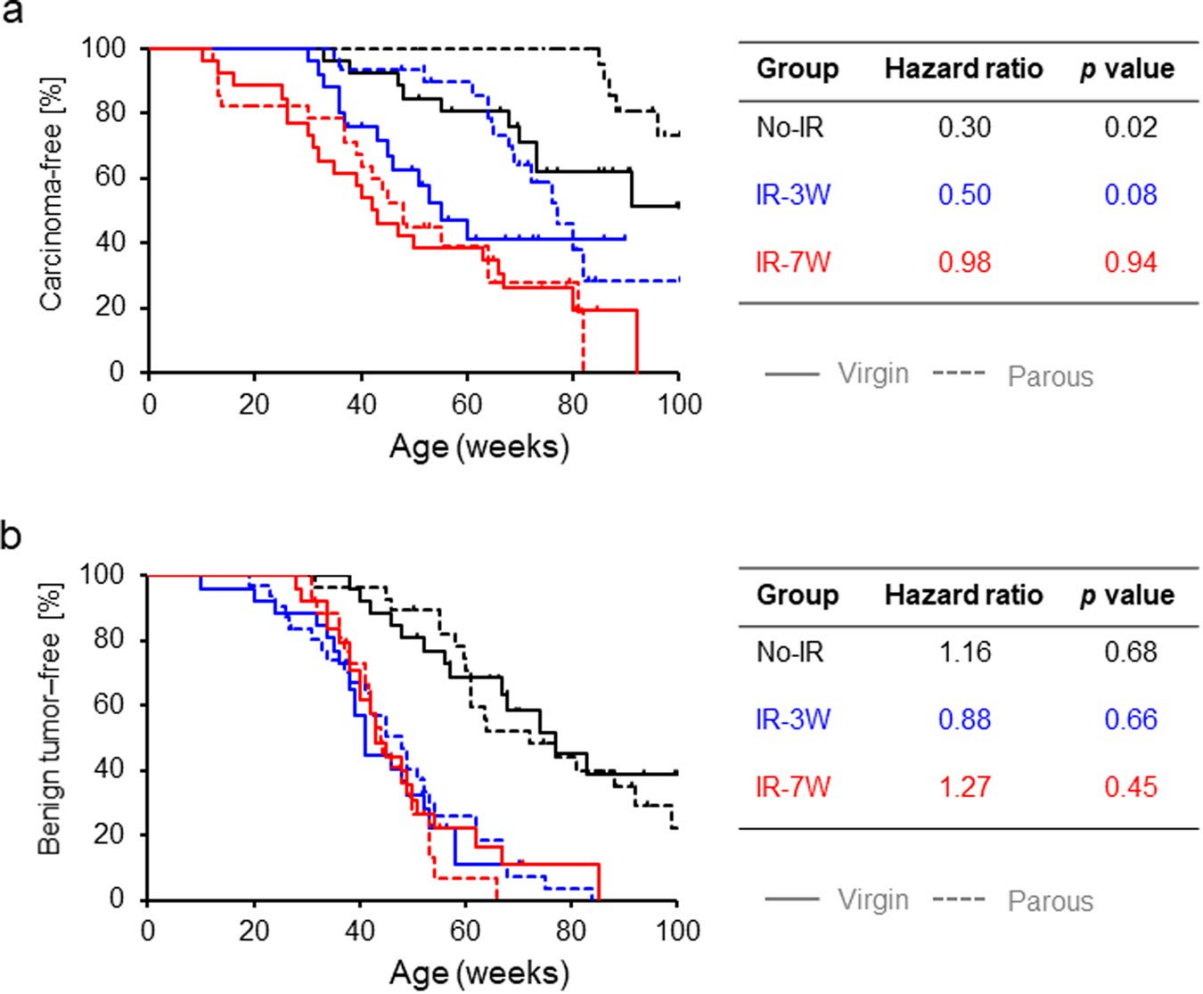
Kaplan-Meier plot for first palpation of a mammary tumor. (**a**) Carcinoma. (**b**) Benign tumors (fibroadenoma or adenoma). *p* values calculated with the log-rank test (virgin vs. parous) are indicated. Hazard ratios for the parous groups are shown, evaluated with the Cox’s model with the respective virgin groups as reference. Solid and dashed lines indicate virgin and parous groups, respectively.

To further assess tumor incidence, time to first palpation of a mammary tumor in each rat (as confirmed retrospectively with a pathology report) was analyzed using the Kaplan-Meier method and Cox’s proportional hazards model. For the No-IR group, parous rats had a significantly decreased and delayed incidence of mammary carcinoma compared with virgin rats (black lines in Fig. 2a). This protective effect of parity offset the risk of pre-pubertal irradiation-induced mammary carcinoma (blue lines in Fig. 2a) but had no effect on the risk induced by post-pubertal irradiation (red lines in Fig. 2a). Despite the similar curves for the IR-7W parous and virgin rats, the relative effect of post-pubertal radiation appeared to be greater for parous rats given their lower rate of spontaneous incidence. In agreement with our previous studies^25–27^, the incidence of carcinoma was higher in IR-7W virgin rats than IR-3W virgin rats (hazard ratio = 1.72, *p* = 0.12). Although the risk of benign tumors was also increased by radiation, parity did not affect the rates of incidence of those tumors (Fig. 2b). Table 1 presents detailed information on mammary tumorigenesis.

### Hormonal status during mammary tumorigenesis

**Figure 3:**
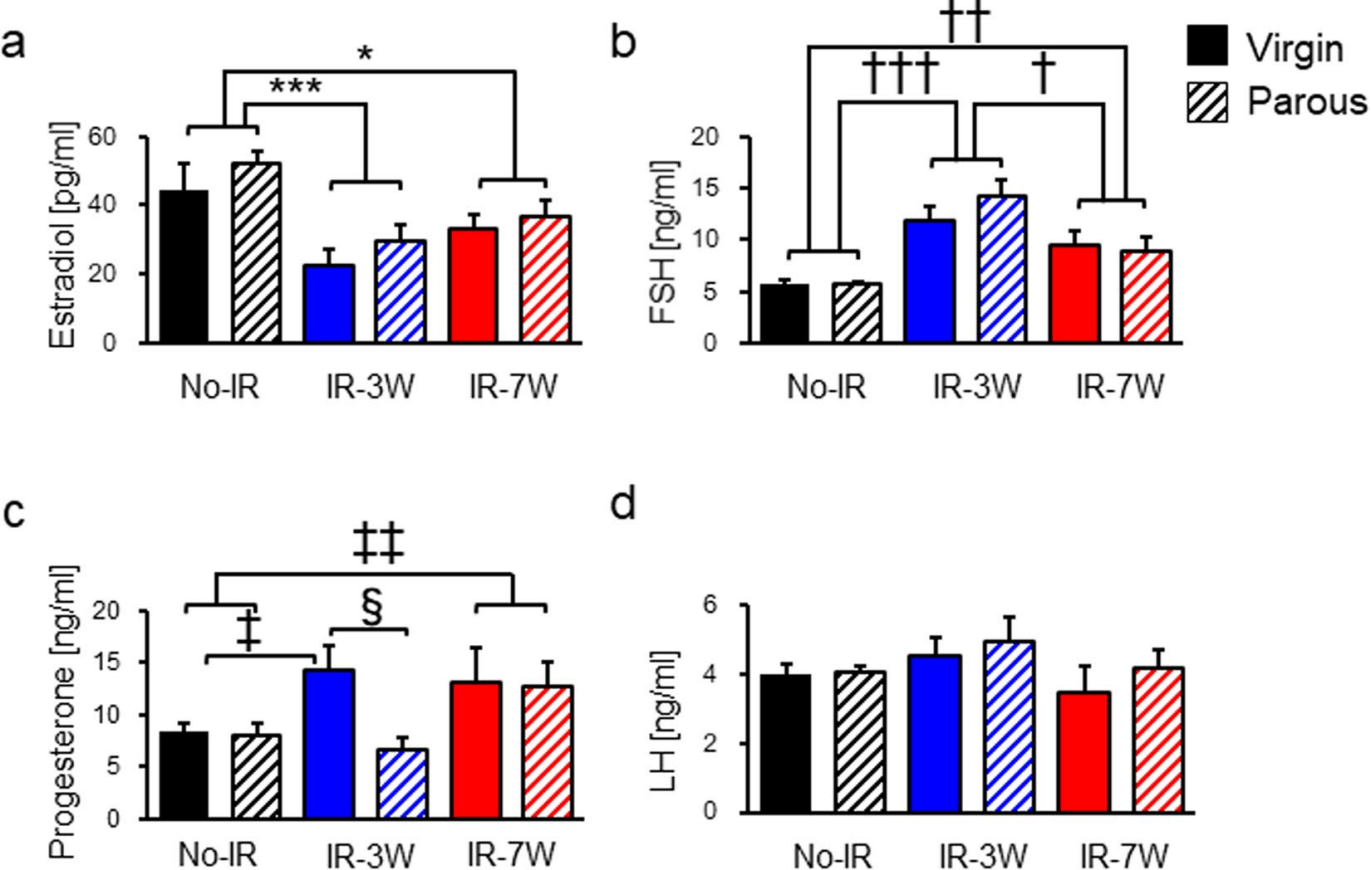
Hormonal status during mammary tumorigenesis. (**a**–**d**) Estradiol, follicle stimulating hormone (FSH), progesterone, and luteinizing hormone (LH), respectively. The number of rats was as follows: No-IR virgin, 8; No-IR parous, 14; IR-3W virgin, 9; IR-3W parous, 8; IR-7W virgin, 5; IR-7W parous, 9. *Student’s *t*-test after 2 × 3 ANOVA; ^†^Welch’s *t*-test after 2 × 3 ANOVA; ^‡^Welch’s *t*-test after 2 × 2 ANOVA; ^§^Student’s *t*-test after 2 × 2 ANOVA. Single, double, and triple symbols indicate *p* < 0.05, <0.005, and <0.001, respectively. Error bars indicate ± SE.

As in previous studies^26,27^, earlier cessation of the estrous cycle was observed here in virgin and parous rats irradiated before but not after puberty (Supplementary Table 1), implying that hormonal alteration associated with radiation-induced ovarian damage depends on the age at exposure. To investigate changes in hormonal status during radiation-induced mammary tumorigenesis, we measured the levels of serum estradiol, progesterone, follicle stimulating hormone (FSH, a pituitary hormone that stimulates ovarian follicle growth), luteinizing hormone (LH, a pituitary hormone that triggers ovulation and development of the corpus luteum), growth hormone, and prolactin (related to mammary tumorigenesis^21,30^) as well as thyroxine and corticosterone (associated with mammary cancer^31,32^); measurements were made between 22 and 26 weeks of age, i.e., after pregnancy and lactation but before the cessation of regular estrous cycling. Mean estradiol level was significantly decreased by radiation exposure regardless of parity or age at exposure (Fig. 3a), whereas mean FSH level showed the opposite trend (Fig. 3b) consistent with a radiation-induced ovarian dysfunction as seen in aging rats^33^. Pre-pubertal irradiation led to a significant increase in mean progesterone level, which was offset by parity; post-pubertal irradiation also increased progesterone level, which remained unchanged by parity (Fig. 3c). This observation is consistent with the previously observed increase in progesterone level in irradiated rats, which was associated with a decreased number of ovarian follicles and increased size of luteinized tissues^34^. In addition, pre-pubertally irradiated virgin rats bearing mammary carcinomas showed a significant increase in progesterone level compared with the tumor-free rats, whereas such an increase was not observed in parous rats (Supplementary Fig. 1). Although temporary elevation of LH in the circulation is required for formation of the corpus luteum and consequent secretion of progesterone, mean LH level was not significantly affected by either radiation exposure or parity (Fig. 3d). Similarly, the levels of other hormones analyzed did not differ significantly among the groups (Supplementary Fig. 2).

### HR status in mammary carcinomas and the effect of parity

**Figure 4:**
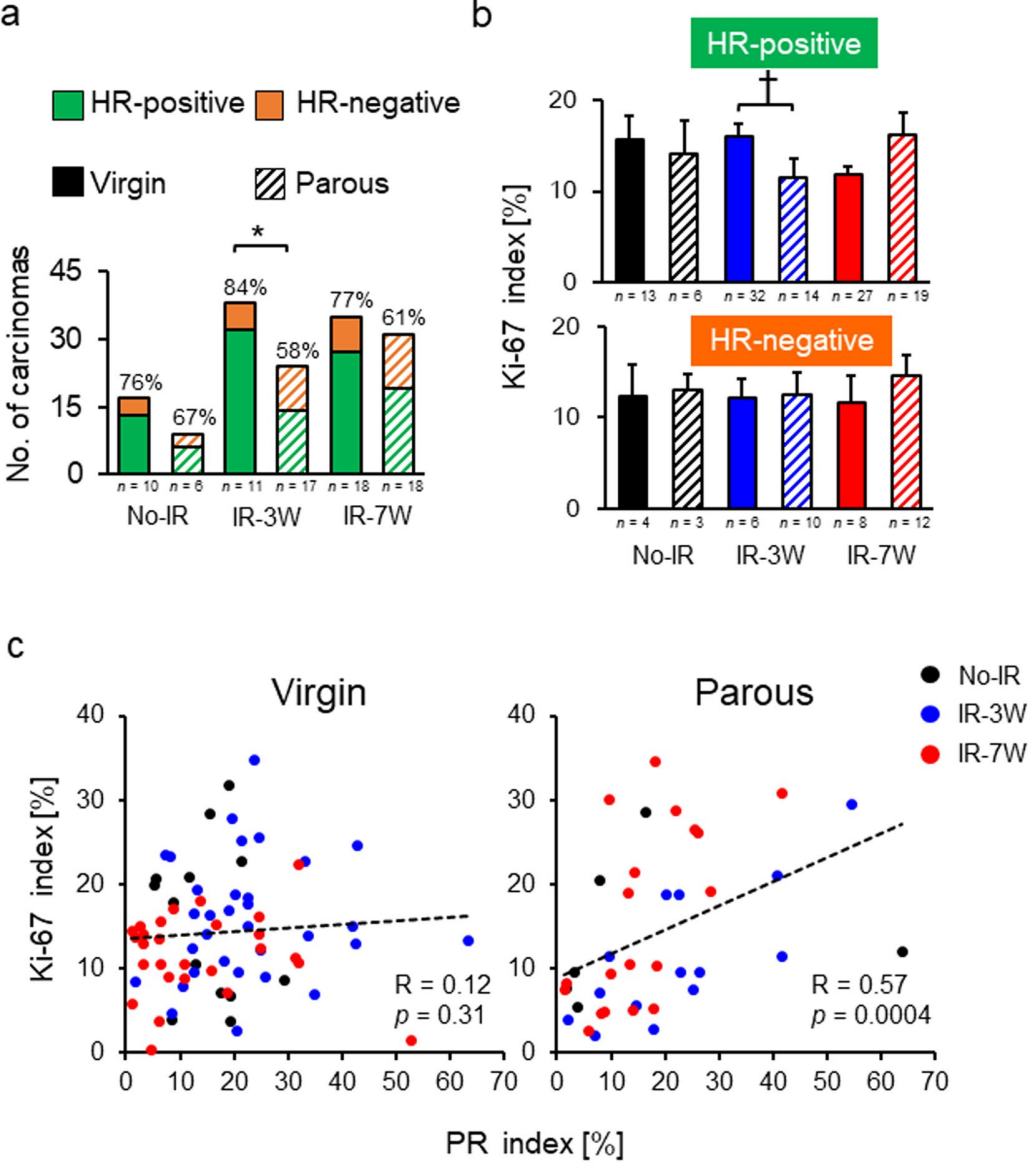
Number and Ki-67 index of hormone receptor–positive and –negative carcinomas, and correlation between progesterone receptor (PR) and Ki-67 expression levels in hormone receptor–positive mammary carcinomas derived from virgin and parous rats. (**a**) The number of carcinomas with positive and negative expression of a hormone receptor (estrogen receptor, progesterone receptor). Percentages indicate the proportion of hormone receptor–positive carcinomas in each group; *n*, number of rats. (**b**) The Ki-67 index for hormone receptor–positive and –negative carcinomas; *n*, number of carcinomas. *p < 0.05 by Fisher’s exact test; ^†^*p* < 0.05 by Mann-Whitney’s *U* test. Error bars indicate ± SE. (**c**) Correlation between PR and Ki-67 expression in hormone receptor–positive carcinomas from virgin and parous rats. The Spearman’s correlation coefficient (*R*) and *p* values are shown in the panels. Circles indicate individual carcinomas.

Epidemiological studies have demonstrated that parity reduces the risk of developing HR-positive mammary tumors^18–20^. To determine how parity modifies the HR status in radiation-induced rat mammary carcinomas, we performed immunohistochemistry for ER and PR as well as the cell proliferation marker Ki-67 to understand their association. Overall, the rat mammary carcinomas were mostly HR positive (virgins, 80%; parous, 61%), yet the percentage of HR-positive mammary carcinomas was significantly decreased by parity in the IR-3W group (84% vs. 58%) (Fig. 4a), a trend not significant in the IR-7W group (77% vs. 61%). For the HR-positive carcinomas remaining in the parous IR-3W group, the Ki-67 was significantly decreased compared with similar tumors in the IR-3W virgin rats. The Ki-67 index was marginally increased (*p* = 0.06) by parity in the HR-positive tumors of the IR-7W group, whereas the Ki-67 index of HR-negative tumors was not changed by parity in any group (Fig. 4b). Thus, parity decreased cell proliferation in HR-positive carcinomas but only in pre-pubertally irradiated rats. Indeed, the delay in age at first palpation by parity seen in the IR-3W and No-IR groups was specific to the HR-positive tumors (Supplementary Table 2). Unexpectedly, irrespective of the age at irradiation, the Ki-67 index was significantly correlated with the PR index in HR-positive mammary carcinomas from parous rats (Fig. 4c; all parous groups combined, *R* = 0.57, *p* = 0.0004; No-IR only, *p* = 0.18; IR-3W only, *p* = 0.0095; IR-7W only, *p* = 0.0029), but not in virgin rats (Fig. 4c). It thus appears that cell proliferation in carcinomas is independent of the PR index in virgin rats, whereas it is related to the PR index in parous rats. The changes in the hormonal status alone (Fig. 3) are unable to fully explain this observation; thus, the results suggest that another mechanism such as hormone responsiveness of the mammary gland is involved in the progesterone-dependent proliferation in HR-positive carcinomas. A similar correlation was not observed between the Ki-67 and ER indices in the HR-positive carcinomas from either virgin or parous rats (Supplementary Fig. 3). Moreover, while the mammary carcinoma cells were strongly stained with PR, normal mammary glands were only weakly stained and no drastic difference was observed between the No-IR and IR groups (irrespective of the tumor-bearing status; Supplementary Fig. 4).

A high frequency of overexpression of human epidermal growth-factor receptor 2 (HER2) has been observed in breast cancer tissue samples obtained from atomic-bomb survivors compared with women who were not exposed to radiation^35^. Although HER2 was overexpressed in approximately 14% of the rat mammary carcinomas in our study, this aspect was not significantly affected by parity or age at exposure (Supplementary Fig. 5).

## Discussion

Our present study reveals a combined effect of the age at exposure and parity on the risk of mammary cancer. The risk induced by pre-pubertal radiation exposure was reduced by parity, whereas the risk induced by post-pubertal irradiation was not affected by parity. IR exposure altered the hormonal environment by reducing serum estradiol and increasing progesterone, which may be associated with ovarian dysfunction, whereas parity normalized the serum progesterone level if it was increased by pre-pubertal IR exposure but not post-pubertal exposure. Parity also seemed to be associated with progesterone-dependent cell proliferation in radiation-induced HR-positive carcinomas. These results suggest that parity differentially affects the onset of radiation-induced rat mammary carcinomas based on the age at exposure via multiple mechanisms. These results suggest that several mechanisms contribute to the differential effects of parity on the onset of radiation-induced rat mammary carcinomas as modulated by age at exposure.

Our results agree with those of previous studies in that parity decreased the risk of spontaneous mammary tumors, as observed in epidemiology studies and models of chemically induced tumorigenesis^8,14^. The present study is the first to demonstrate the preventive effect of parity with respect to the spontaneous development of mammary carcinoma in rats, and this was a consequence of the high frequency of spontaneous mammary tumor development in the present model, which is in agreement with our previous studies^25,26^. In addition, a previous study showed that parity had a negligible effect on the development of mammary carcinomas induced by post-pubertal IR exposure, consistent with the present study, when the rats were irradiated with their ovarian area shielded^16^. The results of the previous and present studies thus imply that the mechanisms underlying the absence of any protective effect of parity after post-pubertal IR exposure is not related to exposure of the ovary. Alternative mechanisms may include alteration in some specific molecular pathways in cancer. For example, pregnancy affords little protective effect for female carriers of *BRCA1*/*BRCA2* mutations^36,37^. Moreover, a study of p53-null mice demonstrated the lack of a parity effect^38^. As these specific genomic aberrations have not been observed in radiation-induced rat mammary carcinoma^39,40^, further determination of altered pathways in mammary cancer will be necessary to fully understand the molecular basis of breast tumorigenesis.

Our results demonstrate that the risk of HR-positive carcinoma is reduced by parity, consistent with epidemiological studies^18–20^. In contrast, the present study did not reproduce the previously reported dependence of the subtype of mammary carcinomas on age at IR exposure^27^; differences between the former study and our study include: (i) a lower radiation dose (2 Gy in the previous study), resulting in a smaller number of carcinomas analyzed; (ii) feeding with a high-fat diet during the experiment; and (iii) a shorter experimental period (until 50 weeks of age vs. 100 weeks in our study). These differences could have contributed to the distinct results concerning carcinoma phenotype. In addition, our observation of the similar frequency of tumors with HER2 overexpression in IR-exposed and non-exposed rats is not consistent with the study of atomic bomb survivors, in which the frequency was higher in exposed women than non-exposed women^35^, but it is consistent with another, more statistically powerful study^41^, in which the frequency was similar.

The interval between IR exposure and pregnancy is a possible factor that influences the protective effect of parity. Epidemiological studies have shown decreased protective effects of parity with increasing age at delivery^42^ that are thought to be attributable to an increase in the number of mutated cells with age^43,44^ and in the proliferation of cells during pregnancy^45^. Because mating was started at a young age of 10 weeks in the present study, pre- and post-pubertal rats had a 7- and 3-week interval, respectively, between IR exposure and mating. Thus, the difference in this interval may have altered the parity-induced protective effect against rat mammary carcinoma. In this regard, a previous study proposed that the frequency of chromosomal translocations in irradiated rat mammary glands does not significantly change with the increase in the interval between IR exposure and analysis^46^. If this scenario applies to our present study, the frequency of genetic aberrations at the time of mating may not differ regardless of the difference in the interval between IR exposure (pre- and post-pubertal) and mating. Further animal and epidemiological studies will be necessary to fully understand the effect of the interval between radiation exposure and pregnancy.

Parity seems to influence mammary carcinogenesis via both systemic and local mechanisms. Rat mammary carcinomas exhibit a hormone dependency that is similar to that of human breast cancer. It has been reported that long-term tamoxifen treatment reduces the incidence of radiation-induced mammary cancer in rats^47^, suggesting that the hormonal environment greatly influences radiation-related mammary cancer risk. In the present study, the pre-pubertal exposure resulted in premature cessation of regular estrous cycling, like that observed for young female patients who received radiation therapy and experienced premature menopause^48^. Because the sensitivity of oocytes to radiation in immature rats is greater than that in adult rats^49^, our results suggest that this differential sensitivity causes differential parity-induced changes in hormonal status. Progesterone stimulates cell proliferation in normal mammary glands^50^ and also in mammary carcinomas^51^. Considering such a general progesterone function for both the normal mammary gland and mammary carcinoma, the increase in serum progesterone as a consequence of radiation exposure may have contributed to the observed increase in the risk of mammary carcinoma. However, it remains unclear why parity after pre-pubertal irradiation normalizes the increased progesterone level. A possible mechanism is related to regression of the corpus luteum after parturition^52^. The high level of progesterone from the corpus luteum during pregnancy is normalized in the postpartum phase, which is related to prolactin-induced corpus luteum regression^53^. The mechanism of the postpartum regression of corpus luteum may be unique in that it is independent of the Caspase-3-mediated apoptosis, whereas the involution in regular menstrual cycles is related with Caspase-3 expression^54^. Such mechanisms may be related to the normalized progesterone levels in the present experiment. Nevertheless, these explanations are hypothetical and further studies are needed. Several studies have reported that parity alters the metabolic function of the mammary gland, liver, and uterus^55–57^, and this may be caused by parity-induced hormonal changes. Further studies of parity-induced systematic and local changes may help clarify the differential effects of age at exposure. In addition to the systemic changes in progesterone level, the present study postulates that parity may be associated with stronger PR dependency of cancer-cell proliferation, as evidenced by the correlation between PR and Ki-67 in the induced carcinomas. The morphology of the post-involution mammary gland in females is distinguishable from that of a virgin^58^. Moreover, several studies have reported that the mammary gland undergoes parity-associated epigenetic modifications, e.g., DNA methylation and histone modifications^59,60^. Because our finding that cell proliferation is altered in mammary carcinoma cannot be readily explained by theories invoking parity-induced changes in hormonal status, further epidemiological and animal studies are needed.

The present study also provides new insights into the observed differences in cancer incidence between virgin rats irradiated at different ages. The risk of mammary carcinomas was lower for IR-3W virgins than IR-7W virgins, with earlier cessation of the normal estrous cycle observed only in IR-3W virgins, reproducing our previous result^27^. That study, however, did not address the effects of hormone levels prior to carcinogenesis. In the present study, estrogen and progesterone levels were comparable among IR-3W and IR-7W virgins at 22–26 weeks of age, which was before the onset of carcinogenesis. Thus, any fluctuations in the levels of these hormones seemingly cannot account for the observed differential effect of age at irradiation, implying that any hormonal changes associated with the cessation of estrous cycles may have had an impact in this regard.

In conclusion, the complex combined effects of parity and radiation exposure on mammary carcinogenesis may include both systemic and local mechanisms. The effect of parity on radiation-induced mammary carcinoma was found to depend on the age at exposure and correlated with serum progesterone level and the responsiveness of the cancer tissue to progesterone.

## Methods

### Animal experiments

All animal experiments were approved by the Institutional Animal Care and Use Committee of the National Institute of Radiological Sciences (approval No. 11–1027 and 16–1030), and were performed in accordance with the Fundamental Guidelines for Proper Conduct of Animal Experiment and Related Activities in Academic Research Institutions under the jurisdiction of the Ministry of Education, Culture, Sports, Science and Technology of Japan. Detailed procedures for the animal experiments are described in our previous reports^25,26^. Briefly, male and female Sprague-Dawley (Jcl:SD) rats were obtained from Clea Japan. Pre- or post-pubertal (3 or 7 weeks of age, respectively) rats were subjected to single, whole-body irradiation (4 Gy from ^137^Cs; 0.5 Gy/min) or left untreated. Rats were palpated weekly for detection of mammary tumors. At age 10 weeks, half of the rats were paired with males; two females were housed together with one male for 2 weeks. The parous rats were allowed to carry a litter to term and to nurse 6 pups for 3 weeks. Mated female rats that failed to wean pups by the time the mothers were 18 weeks old were excluded from the experiment. No significant difference was observed in the weaning rate between the groups (Supplementary Table 1). All rats were fed a CE-2 diet (Clea Japan) throughout the experiment. The estrous cycle for each rat in a subset (*n* = 6 to 9 per group) was monitored for five consecutive days every second week based on the cytology of vaginal smears. Age at cessation of the regular estrous cycle was determined as the age when the cycle was irregular for two consecutive observations. Observation was terminated when rats showed any sign of general deterioration, died, or reached 100 weeks of age. Collected mammary tumors were fixed in 10% neutral buffered formalin, and approximately 4-μm-thick paraffin-embedded sections were stained with hematoxylin and eosin for histological evaluation^15^.

### Measuring hormone concentrations

Rats were grouped as in the aforementioned animal experiment to measure serum estradiol, progesterone, FSH, LH, growth hormone, prolactin, thyroxine, and corticosterone. Five weeks after weaning (age 22–26 weeks), vaginal smears were checked at midmorning. Rats were autopsied between noon and 4 pm on the day of proestrus under isoflurane anesthesia (4% in air). Each serum sample was obtained from blood collected by cardiac puncture and then stored at −80 °C until used. The serum level of estradiol was measured by liquid chromatography-tandem mass spectrometry analysis carried out by the Oriental Yeast Co. Ltd. (Japan) using a liquid chromatography system consisting of a Nexera chromatograph (Shimadzu, Japan) coupled to a SCIEX API 5000 triple-quadrupole tandem mass spectrometer. The electrospray ionization (TurboIonSpray) source was operated in the positive-ion mode to generate estradiol ions. Each serum sample (200 μl) was mixed with 50 μl isotopically labeled estradiol (^13^C_4_, 100 pg/50 μl) as internal controls. The estradiol retention time was 5.11 min. The transitions of estradiol and [^13^C_4_]estradiol (*m/z*) were 544.2/339.0 and 548.2/343.2, respectively. The limit of quantitation was 0.5 pg/μl. Throughout the experiments, the coefficient of variation and recoveries of internal standards were in the range of 0.7 to 8.4% and 82.3 to 103.9%, respectively. The method was validated according to FDA guidance on bioanalytical method validation^61^. Serum progesterone, FSH, LH, growth hormone, prolactin, thyroxine, and corticosterone were analyzed by competitive immunoassays using europium-labeled antibodies prepared in a commercial laboratory (Protein Purify Ltd., Japan). The steroid hormones were extracted with ether (for progesterone) or methanol (for thyroxine and corticosterone), followed by antibody incubations. Serum was diluted at optimal levels, which were between 2- and 60-fold, and then analyzed. The results for FSH, LH, growth hormone, and prolactin are expressed in terms of the NIH rat FSH RP-2, rat LH RP-3, rat growth hormone RP-2, and rat prolactin RP-2 standard preparations, respectively. Measurements were carried out in triplicate, with the intra-assay coefficient of variation (%) and estimated limit of detection (ng/ml) being 3.7%/0.01 (progesterone), 5.9%/0.69 (FSH), 6.0%/0.16 (LH), 7.5%/0.25 (growth hormone), 3.7%/0.11 (prolactin), 3.1%/0.03 (thyroxine), and 6.4%/0.02 (corticosterone). For the measurement of estradiol, progesterone, FSH, and LH, some samples had a very low estradiol level (<10 pg/μl), as expected for proestrus^62^; these samples were considered as outliers as a result of misreading of the vaginal cytology and were thus excluded from further evaluation. The number of excluded samples was as follows: No-IR virgin, 1 sample of 9 total; IR-3W parous, 2 of 10 samples; IR-7W virgin, 2 of 7 samples; and IR-7W parous, 1 of 10 samples. The estradiol and progesterone levels in the No-IR groups were comparable with those reported in a previous study^63^.

### Classification of mammary carcinomas based on HR expression

Formalin-fixed paraffin-embedded tissues were sectioned at approximately 4 μm. Primary antibodies for ER (clone 6F11; Leica, USA; dilution, 1:200), PR (clone PR10A9; GeneTex, USA; dilution, 1:400), Ki-67 (clone SP6; Spring Bioscience, USA; dilution, 1:200), and HER2 (clone e2-4001 + 3B5; Thermo Scientific, USA; dilution, 1:100) were used for immunohistochemistry. After the tissue sections were dewaxed, antigen retrieval was performed by autoclaving the sections at 120 °C for 15 min in 0.1 M tris(hydroxymethyl)aminomethane (pH 8.0), deionized water, and 10 mM sodium citrate buffer (pH 6.0) for ER, PR, and Ki-67, respectively. The tissue sections were treated with 0.3% hydrogen peroxide in methanol at room temperature for 15 min, incubated with 10% normal goat serum (Cedarlane Laboratories, Canada) in a blocking solution (Dako Protein Block Serum-Free; Agilent Technologies, USA) at room temperature for 60 min, and then incubated with a primary antibody at 4 °C overnight. Subsequently, sections were reacted with a peroxidase-conjugated secondary antibody (Histofine SimpleStain MAX-PO (M or R) kit; Nichirei Biosciences, Japan) at room temperature for 60 min. Peroxidase activity was visualized with a 3,3′-diaminobenzidine peroxidase staining kit (SK-4100; Vector Laboratories, USA). Staining for HER2 was performed with an enhancer kit (Super Sensitive™ Polymer-HRP IHC Detection System/DAB, BioGenex, USA) following the autoclaving of the sections in sodium citrate buffer (pH 6.0). Sections were counterstained with hematoxylin after immunohistochemical staining. Stained sections were scanned with a NanoZoomer Digital Pathology system (Hamamatsu Photonics, Japan). Two independent researchers (among M.T., T.I., H.M., and M.N.) randomly chose at least 10 areas (magnification, 40×) from each carcinoma, and then the HR index in the tumor epithelium was measured using Tissue Studio image analysis software (Definiens, Germany). HR-positive tumors were defined as those with positive staining of over 1% of cells for both ER and PR^64^. Tumors were classified as HER2 overexpression (3+, positive) if there was strong membrane staining in 30% or more of tumor cells^65^.

### Statistical analysis

All statistical tests were done using R software (http://www.r-project.org)^66^ with the graphical user interface EZR^67^. Differences in the number per unit time and the age at first palpation of mammary tumors, the number of pups, and the age at the cessation of a regular estrous cycle were analyzed with the Kruskal-Wallis test, followed by pairwise comparisons with the Mann-Whitney’s *U* test. The log-rank test and Cox proportional hazard analysis were performed to analyze the time to first palpation of mammary tumors for each rat. Differences in serum levels of estradiol, progesterone, FSH, LH, growth hormone, prolactin, thyroxine, and corticosterone were evaluated with the multi-way analysis of variance (ANOVA), followed by pairwise comparisons with the Student’s *t*-test or Welch’s *t*-test. The distribution of both tumor subtypes and expression of Ki-67 were compared between virgin and parous groups by the Fisher’s exact test and Mann-Whitney’s *U* test, respectively. Correlations were evaluated by the Spearman’s rank correlation coefficient. A *p* value of less than 0.05 was considered to reflect a statistically significant difference.

## Electronic supplementary material

Supplementary Information
