# Supplementary material for: Differential effect of parity on rat mammary carcinogenesis after pre- or post-pubertal exposure to radiation
Source: Sci Rep. 2018 Sep 25;8:14325. doi: 10.1038/s41598-018-32406-1 (PMC6156598; doi:10.1038/s41598-018-32406-1)
Supplement: Supplementary file 1 — Supplementary Information [file 41598_2018_32406_MOESM1_ESM.docx]

**Supplementary Information**

**Differential effect of parity on rat mammary carcinogenesis after pre- or post-pubertal exposure to radiation**

**Masaru Takabatake, Kazuhiro Daino , Tatsuhiko Imaoka, Benjamin J. Blyth, Toshiaki Kokubo, Yukiko Nishimura, Kaye Showler, Ayaka Hosoki, Hitomi Moriyama, Mayumi Nishimura, Shizuko Kakinuma, Masahiro Fukushi, and Yoshiya Shimada**

**Supplementary Figure 1. Comparison between mean progesterone level of tumor-free rats and rats bearing tumor(s).** Black and dark blue boxes represent rats without and with tumor (s), respectively: *n*, number of rats. ^*^*p* < 0.05 by Student’s *t*-test after 2 × 2 ANOVA. Error bars indicate ± SE.

**Supplementary Figure 2. Serum levels of growth hormone (a), prolactin (b), thyroxine (c), and corticosterone (d).** The number of rats was as follows: No-IR virgin, 3; No-IR parous, 3; IR-3W virgin, 9; IR-3W parous, 10; IR-7W virgin, 7; and IR-7W parous, 10. Error bars indicate ± SE.

**Supplementary Figure 3. Correlation between estrogen receptor (ER) and Ki-67 indices in hormone receptor–positive carcinomas** **derived from virgin and parous rats.** Spearman’s correlation coefficients (*R*) and *p* values are shown in the panels. Circles indicate individual carcinomas.

**Supplementary Figure 4. Representative immunohistochemical images of progesterone receptor (PR) in normal mammary gland and mammary carcinoma.** No-IR NMG, nonirradiated normal mammary glands; IR, Irradiated; HE, hematoxylin and eosin.

**Supplementary Figure 5. Number of mammary carcinomas showing HER2 overexpression and those showing no overexpression in virgin and parous rats.** Percentages reflect the proportion of carcinomas with HER2 overexpression in each group; *n*, number of rats.

| Supplementary Table 1. Changes in reproductive performance after radiation exposure. | | | |
| --- | --- | --- | --- |
| Group | Weaning rate (%) | Number of pups | Age at cessation of regular estrous cycle (weeks) |
| No-IR | 43/50 (85 ± 5^a^) | 14.3 ± 0.5^a^ | Virgin: 36.4 ± 4.4^b^ (*n* = 6) |
|  |  |  | Parous: 38.6 ± 5.4 (*n* = 9) |
| IR-3W | 41/45 (91 ± 4) | 11.9 ± 0.4^*^ | Virgin: 29.5 ± 3.1^†^ (*n* = 6) |
|  |  |  | Parous: 32.3 ± 2.0^‡^ (*n* = 6) |
| IR-7W | 38/43 (88 ± 5) | 14.2 ± 0.4 | Virgin: 34.0 ± 2.4 (*n* = 6) |
|  |  |  | Parous: 35.4 ± 3.6 (*n* = 5) |
| ^a^Standard error; ^b^standard deviation; ^*^*p <* 0.05 vs. No-IR; ^†^and ^‡^*p <* 0.05 vs. No-IR virgin and parous, respectively, by Mann-Whitney’s *U* test after the Kruskal-Wallis test; *n*, number of rats. | | | |

| Supplementary Table 2. Age at first palpation of hormone receptor–positive and –negative mammary carcinomas | | | |
| --- | --- | --- | --- |
| Age at exposure (week) | Parity | Age at first palpation (week) | |
|  |  | HR-positive | HR-negative |
| - | Virgin | 63 ± 14 (*n* = 13) | 48 (*n* = 1) |
|  | Parous | 90 ± 6^*^ (*n* = 3) | 92 ± 8 (*n* = 2) |
| 3 | Virgin | 52 ± 13 (*n* = 16) | 48 ± 16 (*n* = 5) |
|  | Parous | 66 ± 13^*^ (*n* = 9) | 65 ± 15 (*n* = 8) |
| 7 | Virgin | 44 ± 20 (*n* = 21) | 39 ± 20 (*n* = 8) |
|  | Parous | 41 ± 18 (*n* = 16) | 39 ± 30 (*n* = 9) |
| Age at first palpation of carcinoma was compared between the virgin and parous groups. Mean ± SD; *n*, number of carcinomas (excluding postmortem-confirmed non-palpable carcinomas); HR, hormone receptors; ^*^*p < 0.05* by Mann-Whitney’s *U* test. | | | |
